# Maternal obesity in rats results in male-specific increases in genome-wide DNA methylation in postnatal offspring liver

**DOI:** 10.1242/bio.062573

**Published:** 2026-09-01

**Authors:** Neil A. Youngson, Susan M. Corley, Kyoko Hasebe, Aynaz Tajaddini, Michael D. Kendig, Margaret J. Morris

**Affiliations:** ^1^ Department of Pharmacology, School of Biomedical Sciences, UNSW Sydney, Sydney, NSW 2052, Australia; ^2^ School of Biotechnology & Biomolecular Sciences, Faculty of Science, UNSW Sydney, NSW 2052, Australia; ^3^ School of Life Sciences, University of Technology Sydney, NSW 2007, Australia

**Keywords:** Developmental programming, Developmental sex differences, DNA methylation, Liver steatosis, Maternal obesity

## Abstract

Male-specific peripubertal DNA demethylation in the liver has been reported in mice. Here, we investigated whether it also occurs in rats, the influence of maternal obesity and whether DNA demethylation changes contribute to observed sex-specific effects of maternal obesity in offspring. Female rats were fed a high-fat, high-sugar ‘cafeteria’ (Caf) diet before mating with standard chow-fed males. The offspring liver methylome and transcriptome were examined. Body weight was higher in Caf-fed dams prior to mating, during gestation and at parturition. Male and female offspring from Caf-fed dams had lower birth weights but higher adult weights and adiposity than offspring from chow-fed dams. A comparison of DNA methylation in 3-week-old weaner males versus female siblings from chow-fed dams did not reveal the male-specific DNA demethylation that was previously reported in mice. However, strong maternal diet effects in male weaner offspring methylation were observed. A comparison of female weaners from chow- versus Caf-fed dams showed a range of differences, with 39% of differentially methylated regions (DMRs) having higher methylation in Caf offspring and 61% of DMRs having higher methylation in chow offspring. In stark contrast, 99% of maternal-diet-induced DMRs in male weaner offspring had higher methylation in offspring from Caf-fed dams. This suggests that maternal obesity induces widespread hypermethylation in the male offspring liver at weaning. However, a comparison with RNA sequencing data revealed limited transcriptional changes at this developmental stage or in adult offspring. While these data highlight how environmentally sensitive DNA methylation is in the male rodent perinatal period, these methylation changes may not be a major contributor to sex differences in developmentally programmed liver disease.

## Teaser

**Summary:** Maternal obesity has major effects on liver methylation in male but not female offspring; the changes may not affect future liver disease.

## INTRODUCTION

In the last 40 years, the incidence of obesity has increased worldwide (Bluher, 2019). One of the major drivers of the obesity epidemic is thought to be changes in diet, with increased consumption of palatable, ultra-processed foods high in fat and/or sugar, collectively termed western-style or cafeteria (Caf) diets. One consequence of the obesity epidemic is the corresponding rise in obesity in women of childbearing age, which is now known to impact offspring development, growth and disease risk (Vahratian, 2009). Human and animal studies have shown that prenatal, perinatal or postnatal maternal obesity can lead to increased offspring birth weight and adiposity, altered glucose metabolism, hypertension and dyslipidaemia, increasing the risk of metabolic diseases [type 2 diabetes (T2D), fatty liver disease], neurological diseases and certain cancers. Several studies, including work from our lab, have highlighted differences in maternal-diet-induced responses between male and female offspring; however, the mechanistic underpinnings of these differences are poorly understood (Matuszewska et al., 2021; Bahari et al., 2013).

The liver has been frequently studied in maternal obesity given its influential role in energy metabolism and metabolic disease. It undergoes extensive developmental changes in the period from late gestation to weaning, when offspring are exposed to maternal diet through the placenta or via milk. During gestation, the liver is a primarily haematopoietic organ that mainly utilises carbohydrates for energy; around birth, the haematopoietic cells are replaced by liver parenchymal cells (Cannon et al., 2016), and the organ gains importance for offspring energy metabolism, mainly utilising lipids from milk as an energy source (Hashimoto and Ogawa, 2018). At weaning, liver energy utilisation changes yet again towards carbohydrates as well as lipids in preparation for an adult diet (Hashimoto and Ogawa, 2018; Ehara et al., 2015). This dynamic period for energy metabolism is accompanied by large-scale changes in hepatic gene expression and DNA methylation (Cannon et al., 2016) and has been proposed to be sensitive to the macronutrient composition of the mother's diet. Indeed, ‘programmed’ responses in the liver have been shown to persist into adulthood, increasing the risk of diseases, especially fatty liver disease (Savva et al., 2022; Alfaradhi et al., 2014; Thompson, 2020).

It was recently discovered that, in addition to the hepatocyte differentiation-associated DNA methylation changes post-birth (Cannon et al., 2016), there is a genome-wide reduction in DNA methylation in male, but not female, mouse livers in the period between embryonic day (E)18.5 and adulthood (∼6 weeks old) (Reizel et al., 2015). These changes are found at thousands of regions, including genes and enhancer regions (Reizel et al., 2015), and are regulated by testosterone-induced TET demethylase enzymes (Reizel et al., 2018). This demethylation has been shown to alter many hepatocyte functions, including increasing the expression of genes required for the increased oxidation of lipids from milk (Ehara et al., 2015; Reizel et al., 2018). Indeed, it has been proposed that cellular differentiation in development itself may be facilitated by a transition from a relatively transcriptionally repressive genome state of the early embryo to an increasing level of genome demethylation and gene activation (Cedar et al., 2022).

In this study, using an established rat model of maternal obesity (Raipuria et al., 2015; Tajaddini et al., 2022), we aimed to investigate whether the male-specific postnatal DNA demethylation is affected by maternal consumption of an obesogenic Caf diet and whether it could be important for the observed sex differences in offspring physiological responses to maternal diet (Rodriguez-Gonzalez et al., 2019; Ayonrinde et al., 2018; Tajaddini et al., 2022).

## RESULTS

Caf diet increased dam body weight by 12% prior to mating, although the body and liver weights of the two groups were comparable at euthanasia (Table 1). Caf-fed dams had larger litter sizes, and their male and female pups had lower body weights than pups from chow-fed dams. At weaning, female offspring from Caf-fed mothers had higher body weights than females from chow-fed mothers. However, at 14 weeks of age, the group body weights were not different. The opposite pattern was seen in male offspring, with significant body weight increases in offspring of Caf-fed mothers in adulthood, but not at weaning (Table 1). Liver mass was significantly higher in male and female offspring of Caf-fed mothers at weaning, but not in adulthood. Increased liver lipid content may contribute to these changes, as the liver scores were higher in male and female weaners from Caf-fed mothers, but not in adulthood. At weaning, whole-body fat mass was significantly increased in male offspring of Caf-fed dams compared to male offspring of chow-fed dams. A similar but not significant trend was seen in female siblings (Fig. S1).

**Table 1.** Top: maternal pre-mating body weight and offspring birth weight plus litter size. Bottom: body weight, liver mass and liver score of chow- and Caf-fed mothers and their 3- and 14-week-old offspring (one representative of each sex per litter)

	Pre-mating BW		Day 1 female offspring BW		Day 1 male offspring BW		Litter size	
	Chow-fed dam	Caf-fed dam	Chow pup	Caf pup	Chow pup	Caf pup	Chow	Caf
Body weight (g)	293.9±3.9	343.4.1±13.4**	6.6±0.1	6.2±0.1**	7.1±0.1	6.3±0.1***	12.6±0.7	14.6±0.6*

			Female weaner offspring		Female adult offspring		Male weaner offspring		Male adult offspring	
	Chow-fed dam	Caf-fed dam	Chow mother	Caf mother	Chow mother	Caf mother	Chow mother	Caf mother	Chow mother	Caf mother
Final body weight (g)	386.9±7.2	404.1±8.4	41.0±1.2	46.1±1.4**	292.9±7.7	303.3±11.4	41.7±1.8	44.8±0.9	521.5±20.1	594.3±26.7*
Liver mass (g)	16.83±0.76	15.59±0.42	1.49±0.06	1.74±0.06**	9.78±0.55	9.70±0.48	1.48±0.07	1.69±0.06*	18.35±1.56	20.85±1.46
Liver score	0.63±0.18	0.63±0.18	0.75±0.25	2.00±0.06***	0.25±0.16	0.38±0.18	0.63±0.42	1.88±0.40*	0.75±0.15	1.25±0.37

Day 1 body weights (BWs) are mean±s.e.m. of eight individual litters. Data are shown as mean±s.e.m., *n*=8 per group. To explore maternal diet effects, data were analysed by two-tailed Student's *t*-test or two-tailed Mann-Whitney *U* test for liver score. *P<0.05, **P<0.01, ***P<0.001 maternal diet effect.

### RNA sequencing (RNA-seq) reveals maternal-diet-associated gene expression changes in offspring at weaning but not adulthood

Livers from dams and offspring collected at weaning and adulthood underwent RNA-seq analysis. Multidimensional scaling (MDS) plots demonstrated that gene expression differed most strongly between weaners and dams/adults (Fig. S2). Additionally, marked sex differences were observed in adults, with dams grouping with their adult female offspring and away from adult male offspring (Fig. S2A). MDS plots of just the offspring also show stronger effects of sex and age than maternal diet, although the sex effects were less pronounced in weaners than adult offspring (Fig. S2B).

Comparing the livers of chow- and Caf-fed dams identified 1458 differentially expressed genes (DEGs) (622 lower and 836 higher in Caf-fed rats) (Table 2; Dataset 1). KEGG analysis of the genes with higher transcript levels in Caf-fed dams revealed that many metabolic pathways were significantly enriched, particularly those involving lipid metabolism, amino-acid metabolism and glycolysis/gluconeogenesis (Dataset 1). Examination of the genes that were lower in Caf-fed dams revealed significant enrichment of inflammation and immune pathways.

**Table 2.** Numbers of DEGs from RNA-seq analysis from dams and offspring comparisons

Group comparison	DEGs higher in the first group	DEGs higher in the second group
Chow-fed dam versus Caf-fed dam	622	836
Female weaners from chow-fed dams versus female weaners from Caf-fed dams	80	180
Male weaners from chow-fed dams versus male weaners from Caf-fed dams	53	90
Adult female offspring from chow-fed dams versus adult female offspring from Caf-fed dams	3	8
Adult male offspring from chow-fed dams versus adult male offspring from Caf-fed dams	21	15

All adjusted *P*<0.05.

Examination of the transcriptomes of female and male offspring livers at weaning suggested that maternal diet impacted several physiological responses. Comparison of female weaners from the two maternal diet types identified 260 DEGs, of which 80 were lower, and 180 were higher in offspring of Caf-fed dams than in offspring of chow-fed dams, respectively (Table 2; Dataset 1). The same comparison in male weaners identified 143 DEGs, of which 53 were lower and 90 were higher in offspring of Caf-fed dams (Table 2; Dataset 1). There were 99 DEGs in common between males and females (24 downregulated and 75 upregulated).

**Fig. 1.**
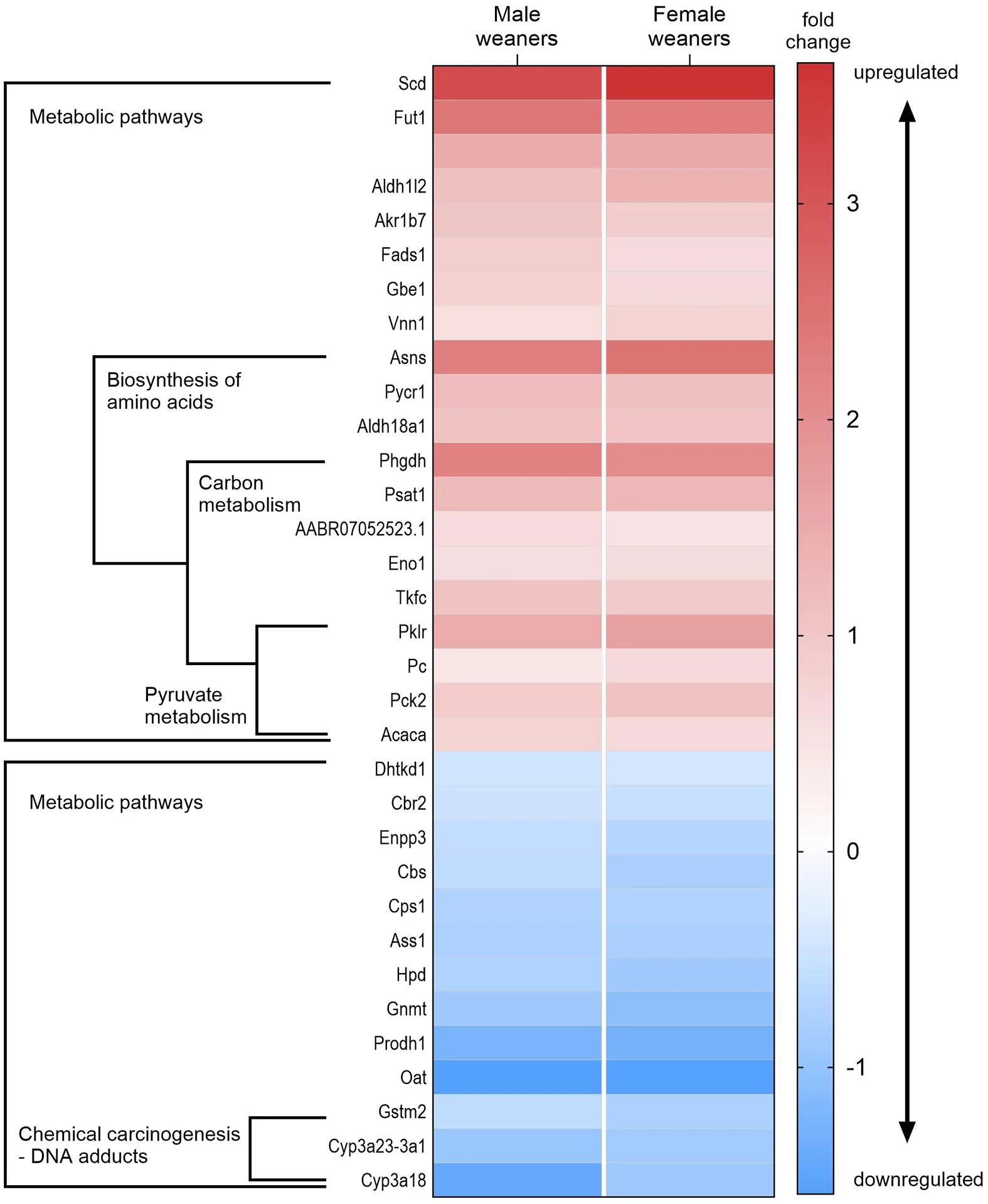
**Heatmap showing the most significantly enriched KEGG pathways that were commonly upregulated or downregulated by maternal obesity in both male and female weaner offspring based on liver RNA-seq.** Selected DEGs in each pathway are displayed.

KEGG analysis of DEGs revealed that overall male and female weanling offspring exhibited similar responses to maternal Caf diet (Fig. 1; Dataset 1). Fig. 1 shows a heatmap of representative genes and pathways similarly significantly affected by maternal diet in male and female weaner offspring. When first considering the genes with higher levels in Caf-fed dams, male and female offspring exhibited the same top four enriched pathways: biosynthesis of amino acids, carbon metabolism, pyruvate metabolism, and metabolic pathways. Similarly, all of the significantly enriched pathways from the DEGs which were lower in female offspring from Caf-fed dams were also significant in male offspring, including metabolic pathways, chemical carcinogenesis (DNA adducts), steroid hormone biosynthesis, retinol metabolism and drug metabolism. The few differences between the male and female weaners' responses to maternal Caf diet were that, in males, DEGs with higher levels in Caf diet group showed significant enrichment of fatty acid metabolism, whereas in females, DEGs with lower levels in offspring from Caf-fed mothers showed enrichment of other pathways such as glutathione metabolism and fluid shear stress and atherosclerosis.

A comparison of female and male offspring livers in adulthood revealed fewer differential responses to maternal diet than at weaning. Female offspring had 11 DEGs (three lower, eight higher in offspring of Caf-fed rats, respectively) (Table 2; Dataset 1). Male offspring had 36 DEGs (21 lower, 15 higher in offspring of Caf-fed rats, respectively) (Table 2; Dataset 1). Only two genes were differentially expressed in both male and female adult offspring. *EF-hand calcium binding domain 6* (*Efcab6*) was higher in both male and female offspring of Caf-fed rats, whereas *RGD15602* was higher in offspring of Caf-fed rats in males but was lower in females. KEGG analysis revealed no enriched pathways in male or female adult offspring of obese mothers.

### Reduced representation bisulphite sequencing (RRBS) of weaner livers reveals widespread higher methylated regions in male offspring of Caf mothers compared to offspring of chow mothers

Sex differences in postnatal liver demethylation have previously been described only in mice (Reizel et al., 2018; Reizel et al., 2015). Therefore, we compared male and female weaner-stage DNA methylation in offspring from control diet mothers. Due to random inactivation of one X-chromosome in females, the 913 (*P*<0.05) differentially methylated regions (DMRs) included 660 regions with higher methylation in the female X-chromosome (Dataset 1, Fig. S3). However, of the autosomal DMRs, 60 also had higher methylation in females, whereas 191 had higher methylation in males. This pattern is not consistent with the male-specific genome-wide demethylation described in mice (Reizel et al., 2018; Reizel et al., 2015). These X-chromosomal and autosomal methylation differences were also seen in a comparison of male and female weaners from Caf-fed mothers (Dataset 1, Fig. S3).

**Fig. 2.**
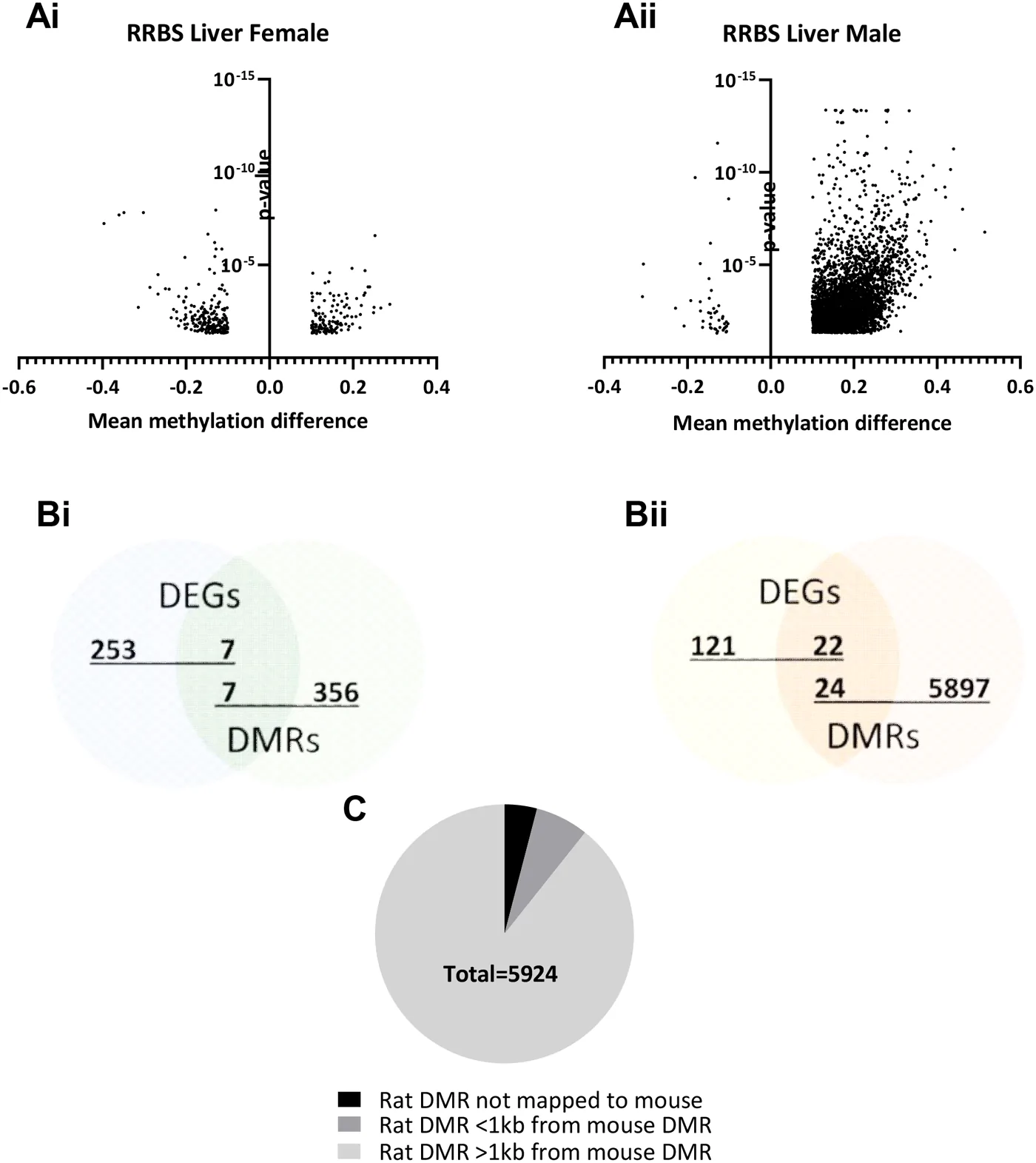
**Analysis of the offspring liver DMRs.** (A) Volcano plots of RRBS DMRs between female [(i) *n*=4 versus 4] and male [(ii) *n*=5 versus 5] weaner offspring livers from chow versus Caf mothers. Only DMRs with >10% methylation group difference (*P*<0.05) are displayed. In females, similar amounts of DMRs had increased or decreased methylation in offspring from Caf-fed mothers. In males, 99% of all DMRs had higher methylation in offspring from Caf-fed mothers. (B) Venn diagrams showing comparisons of DEGs from RNA-seq and DMRs from RRBS in female (i) and male (ii) offspring livers. An overlap indicates that DMR is within 10 kb of the DEG. Different numbers in the overlap region between DEGs and DMRs relate to a gene having more than one DMR or a DMR being within 10 kb of two genes. (C) Comparison of 5924 (*P*<0.05) DMRs between the male rat offspring liver of chow- and Caf-fed mothers, and 52,025 regions that undergo demethylation postnatally in male mouse liver (Reizel et al., 2018).

Locus-specific CpG methylation analyses of same-sex weaner livers revealed DMRs in comparisons between offspring from chow- and Caf-fed dams. A comparison of four chow and four Caf female offspring identified 363 DMRs (Fig. 2Ai; Dataset 1). Of these, 223 (61%) had lower methylation and 140 (39%) had higher methylation in Caf offspring than in chow offspring, respectively. In contrast, a comparison of five chow and five Caf male offspring identified 5924 DMRs (Fig. 2Aii; Dataset 1), of which only 45 (1%) had lower methylation and 5879 (99%) had higher methylation in Caf offspring than in chow offspring. The greater number of DMRs in males is unlikely to be due to greater statistical power (there were five in each group, compared to four in the female groups), as a four-versus-four comparison of male samples identified 2184 DMRs, still far exceeding the 363 in females. The extreme difference in the proportion of DMRs, with 99% having higher methylation in Caf offspring, is also unaffected by the group size difference.

The 100 most significant DMRs in male weaners (all *P*<6×10^−9^, with an average methylation difference of 23% higher methylation in offspring of Caf-fed dams, range 18% lower to 44% higher) were at or nearby 69 genes. Although no significant KEGG enrichment was seen with these genes, there were notably seven genes which undergo genomic imprinting (*Nnat*, *Blcap*, *Grb10*, *Kcnq1*, *Zrsr1*, *Igf2r* and *Gnas*). The 100 most significant DMRs in female weaners (all *P*<0.0038, with an average methylation difference of 4% lower methylation in offspring of Caf-fed dams, range 40% lower to 29% higher) were at or nearby 50 genes. As in males, no KEGG pathways were significantly enriched, and the list contained some imprinted genes (*Bmp7*, *Grb10*, *Gnas*, *Begain*).

An examination of the genomic elements which are located at the DMRs in each group comparison is summarised in Table S1. This revealed differences in the proportions of DMRs which were located at or near transcriptional start sites (TSS), CpG islands (CGIs), repetitive elements and liver-specific active or primed enhancers. It is particularly noted in this analysis that the regions of the genome with increased methylation in male offspring due to maternal Caf diet included all genomic element types, but with the greatest abundance in TSS and repetitive elements. The sex-specific DMRs were particularly common at TSS and CGIs on the X-chromosome, as is to be expected with X-chromosome inactivation.

### DMRs at weaning do not underlie the majority of transcriptional differences in weaner and adult offspring livers

In female weaner livers, only seven of the DEGs had DMRs within 10 kb (Fig. 2Bi). However, two of the seven had been previously linked to maternal obesity. Hepcidin (Hamp) is a key regulator of circulating iron levels. A DMR in the intron of Hamp had 11% higher methylation in weaners from Caf-fed dams, and the transcript was the DEG with the largest negative fold change, supporting its downregulation due to maternal Caf diet. A human study has shown that maternal overweight or obesity is negatively correlated with neonatal (umbilical cord blood) iron and hepcidin levels (Jones et al., 2021). Similarly, the gene with the third-largest positive fold change difference between the groups, fibroblast growth factor 21 (Fgf21), had a corresponding 17% decrease in methylation at its promoter. Fgf21 is a liver-secreted peptide hormone, which has potent direct regulatory effects on energy metabolism in various tissues. It has been identified as a mediator of the effects of maternal obesity on offspring in multiple rodent studies, though sometimes with increased (Moeckli et al., 2022) and sometimes reduced (Shankar et al., 2010) expression. Increased DNA methylation due to maternal high fat diet (HFD) at the Fgf21 promoter in the offspring liver was reported in a mouse study, but it did not assay for transcriptional effects associated with the methylation change (Wankhade et al., 2017).

In male weaners, 22 of the DEGs had DMRs within 10 kb (Fig. 2Bii). KEGG analysis of these 22 genes revealed significant enrichment for pathways involved in mitochondrial respiration and glucose, lipid and amino-acid metabolism. Of these genes, only Fads1 has been implicated as a mediator of changes in the offspring liver due to maternal obesity (Kelly et al., 2022). However, in contrast to the previous study, Fads1 was increased in offspring from Caf-fed dams in this study.

A comparison of the DMRs observed at weaning to the DEGs in adult siblings revealed that none of the 11 adult female DEGs had DMRs within 10 kb of the same gene in livers of offspring at weaning. Similarly, of 36 genes with differential expression in adult males due to maternal diet, only four, *Clstn1*, *Slc30a2*, *Togaram2* and *Zbtb7c*, had DMRs within 10 kb in male weaners, and none was differentially expressed at weaning. *Zbtb7c* is a key regulator of gluconeogenesis in response to fasting in mice (Choi et al., 2019).

Pearson correlation was undertaken to examine the relationship between methylation and transcription at DEGs with DMRs within 10 kb (Table S2). This analysis does identify a few genes with strong associations of high methylation/low expression or vice versa, such as *Hamp* and *Fgf21* in female weaners. The Pearson correlation coefficient for these seven genes is strong at 0.85 but drops to a negligible 0.005 when *Hamp* is excluded, revealing that it is driving the group correlation. However, for males, in which 99% of DMRs have higher methylation in offspring of Caf dams, the 22 genes with nearby DMRs had only a weak Pearson correlation coefficient of 0.24. Only four DEGs in adult males had a nearby DMR, and these had a moderate Pearson correlation coefficient of 0.5. Therefore, the genome-wide DNA methylation increases in weaner male offspring of Caf-fed dams compared to chow-fed dams appear to not have caused a general decrease in gene expression.

The disconnect between methylation and expression is also illustrated in Fig. S4, which shows similar expression patterns of genes in males and females, in spite of significant differences in nearby DMRs at weaning.

### Comparison of DMRs to regions undergoing postnatal demethylation in mouse liver

Whole-genome bisulphite sequencing in male mouse liver by Howard Cedar's group at the Hebrew University Medical School, Israel, identified 52,025 regions that undergo demethylation postnatally (Reizel et al., 2018). We compared the 5294 (*P*<0.05) DMRs between male rat offspring from chow- and Caf-fed mothers to these mouse DMRs and identified 394 which were within 1 kb of the orthologous genomic regions (Fig. 2C; Dataset 1). KEGG analysis of the genes nearest the 394 common DMRs identified 13 pathways with *P*<0.05. None of the pathways passed correction for multiple testing, but it was notable that nine of the 13 were disease associated, including cancers (gastric, leukaemia and lung), alcoholic liver disease, non-alcoholic liver disease, Cushing's syndrome and spinocerebellar ataxia.

## DISCUSSION

Our rat cohort mirrors many key aspects of pathophysiology of increased energy intake and associated obesity during pregnancy in terms of the effects on maternal and offspring outcomes. As expected, due to its higher caloric content and palatability, the Caf diet increased the pre-mating body weight of dams (Leigh et al., 2019). The Caf diet's effects on maternal liver transcriptomes were profound, with 103 significantly enriched KEGG pathways identified when examining the DEGs with higher or lower transcript levels in the Caf group. The alteration of processes involved in energy metabolism was particularly evident, with 13 of the significantly enriched pathways in the genes with higher levels in the Caf being linked to these. The predominance of immunity and inflammation KEGG pathways in the genes expressed at lower levels in the Caf group was somewhat unexpected but may be linked to diet-induced differences in postnatal liver involution (Goddard et al., 2017).

The phenotypic effects of maternal obesity on offspring were also consistent with previous rodent studies, with reduced birth weight (Sanches et al., 2022) yet increased body weight in later life (female weaners and/or male adults) (Menting et al., 2019; Tajaddini et al., 2022). Liver weights and liver scores, an indicator of liver lipid content, were higher in female and male weaners born to Caf-fed dams, also frequently reported in maternal obesity studies (Rodriguez-Gonzalez et al., 2019; Tajaddini et al., 2022). Overall, the transcriptional effects of maternal diet were strikingly similar between males and females, with major impacts on metabolic pathways, which could explain the increased liver weight and liver score in weanlings from Caf-fed dams. An increase in liver sensitivity to maternal diet in males (Savva et al., 2022) was also evident in the higher number of DEGs in adult males than in females. Transcriptomics indicated a much lesser impact of maternal diet on adult offspring than in weaners, likely resulting from postweaning exposure to a healthy (chow) diet for 11 weeks. This lessening of impact has been reported for body and organ weights, adiposity, glucose tolerance, gut microbiota, and transcriptomics (Yadav et al., 2025; Kendig et al., 2023), particularly in young to middle-aged offspring. Importantly, however, effects of maternal obesity tend to return in later age or if the offspring receive unhealthy diets themselves (Rodriguez-Gonzalez et al., 2019; Uddin et al., 2017).

This study is the first to report the considerable difference in maternal-diet-induced genome-wide DNA methylation changes between male and female offspring (Fig. 2). In female offspring, the similarity in the proportion of regions where methylation increased and decreased is the most commonly seen pattern for any change in cell state, as it mirrors the combination of genes which are epigenetically activated or repressed. In male offspring, almost all DMRs had higher methylation levels if they had a Caf-fed mother than if they had a chow-fed mother. This type of extreme bias is sometimes seen when epigenetic modifier genes are mutated experimentally (in cells of animal models) (Bourc'his and Bestor, 2004; Daxinger et al., 2013) or in diseased cells (such as cancer cells) (Assante et al., 2022; Chen et al., 2022). However, they are rarely seen *in vivo* in viable, healthy mammals.

We originally hypothesised that male and female offspring from chow-fed dams would predominantly have DMRs that had less methylation in males than in females, as this is what was reported in mice. However, this was not observed. There were relatively few DMRs, and most were on the X-chromosome, reflecting the increased DNA methylation on the inactive X-chromosome in females. This may reflect a species specificity of male postnatal DNA methylation in the liver or could suggest that it occurs at a different timepoint in rats. In mice, the demethylation mainly occurs between 3 and 6 weeks after birth (Reizel et al., 2018), so it may not have initiated in the 3-week-old rats used in this study. Instead, our data suggest that, in rats, maternal Caf diet increases DNA methylation levels at thousands of regions throughout the genome. Further evidence of the difference between the mouse and rat studies is that relatively few of the rat DMRs were within 1 kb of the mouse regions which undergo postnatal demethylation (Fig. 2C).

Many previous studies in rats (Rodriguez-Gonzalez et al., 2015; Jacobs et al., 2014; Raghavendhira et al., 2025) and humans (Laru et al., 2024; Negri et al., 2026) have observed reduced testosterone in male offspring from obese or overweight mothers and indeed fathers (Sanchez-Garrido et al., 2018; Raghavendhira et al., 2025). The male-specific postnatal liver demethylation in mice is regulated by testosterone activating the DNA demethylase TET2 (Reizel et al., 2015). Therefore, it is possible that the increase in DNA methylation in the male offspring of Caf-fed mothers in this study is due to low testosterone. A recent mouse maternal obesity study that characterised liver histone methylation (H3K9met3) in offspring at weaning did not identify such a pronounced difference in the abundance of DMRs between male and female offspring (Yadav et al., 2025). Therefore, perhaps the epigenetic sex differences are limited to DNA methylation due to the testosterone TET2 mechanism.

A major question that arises from this discovery is as follows: just how important is perturbation of the male-specific postnatal methylation processes for the physiological effects of maternal obesity at weaning and in later life? Future work is needed to identify whether these differences persist or resolve in adulthood. However, insight into this question was given by comparing the DMRs with DEGs in the same tissues. Considering that there were more than ten times the number of DMRs in male than female offspring, it is surprising that there were fewer DEGs in males than in females. This may indicate that the large-scale impact of increased methylation does not necessarily lead to large-scale effects on liver function. The high proportion of DMRs that contained repetitive elements and the relatively few DMRs that are in liver enhancers may indicate that a large proportion of DMRs are in non-functional regions of the genome. Furthermore, the similarity in overall maternal-diet-induced transcriptional changes in males and females suggests that impacting the male-specific DNA methylation is not a major mediator of the physiological changes associated with maternal obesity.

However, some caution is needed in this general interpretation. Firstly, there were a small number of genes which are influential for liver metabolic function and had either differential methylation or differential methylation and expression. Of the 22 genes which had DMRs and were differentially expressed in male offspring (Fig. 2Bii), several are of importance for the metabolism of glucose (*Dhtkd1*, *Eno1-ps20*, *Pklr*, *Pc*, *Sds*), lipid (*Fads1*, *Mogat3*, *PC*, *Sds*) and amino acids (*PC*, *Pklr*, *Pycr1*). The presence of seven imprinted genes within 10 kb of the top 100 most significantly methylated DMRs was also intriguing considering the role of those genes in the regulation of postnatal growth and metabolism (Millership et al., 2019).

Secondly, the potential for latent effects of the methylation changes at weaning cannot be ruled out. If they persist into adulthood, they could influence age-related physiological changes, the response to additional challenges on the liver (e.g. introduction of an unhealthy diet in offspring or fasting) or chronic diseases where the liver is of clinical importance [such as metabolic dysfunction-associated steatotic liver disease (MAFLD), T2D]. For example, in a mouse model of maternal-diet-induced obesity, 12-month-old male offspring from obese mothers had liver steatosis and overexpression of the key fibrosis gene collagen *Col1a1* (Mennitti et al., 2022) compared to males from control diet mothers. In our rat study, the promoter of *Col1a1* had significantly lower methylation at its promoter in male weaner offspring livers from Caf mothers than from chow mothers (Dataset 1), yet there was no associated transcriptional change at this earlier stage (14 weeks old in this study versus 52 weeks old in the mouse study). If latent consequences of the methylation changes are the case, then they would invite research on preventive therapies which aim to correct the maternal-diet-induced methylation changes to reduce disease risk. The genes enriched in disease-associated pathways which undergo postnatal demethylation in male mice (Reizel et al., 2015) also were DMRs in the male rat offspring in this study (Fig. 2C, Dataset 1), supporting the relevance of these genomic regions for later-life disease. Additionally, a recent study in non-human primates identified maternal-obesity-induced metabolic changes in the liver, which differed in later life between male and female offspring (Ampong et al., 2022).

In conclusion, our study highlights the sensitivity of genome-wide DNA methylation in the male postnatal liver to maternal diet. However, our data suggest that the methylation changes are not responsible for the majority of gene transcription changes that are induced by the maternal Caf diet. Nonetheless, the correlation of DNA methylation and transcriptional changes at a small number of influential genes and the potential for latent effects indicate that further investigation is needed to evaluate the importance of the pervasive DNA methylation changes for health and disease in later life.

## MATERIALS AND METHODS

### Animal procedures

Young adult female (approximately 7-8 weeks of age; body weight ∼200 g) and male (approximately 8-9 weeks of age; body weight ∼300 g) Sprague-Dawley rats were obtained from a commercial supplier (Animal Resource Centre, WA, Australia). They were housed four per cage in a colony room maintained at 18-22° (12-h light/dark cycle). Water and standard chow (14 kJ/g, 65% carbohydrate, 22% protein and 13% fat; Premium Rat Maintenance diet, Gordon's Stock Feeds, NSW, Australia) were continuously available. Following acclimatisation, female rats were weight matched and then randomly allocated to receive chow (*n*=10) or Caf (*n*=15) diet, consisting of a selection of cakes, biscuits and protein sources (e.g. meat pie and dim sims), that varied daily, with chow and water always available, as described previously (Leigh et al., 2019). The Caf-style diet is high in fat and sugar (Leigh et al., 2019), which leads to a more robust obesity phenotype than purified homogeneous high-fat diets (Buyukdere et al., 2019; Sampey et al., 2011). After 6 weeks of diet, females were mated with chow-fed males by co-housing two females and one male for 5 days. The male was then removed. Pregnancy was inferred based on weight gain, and females were housed individually from approximately gestation day 16. Litters were standardised to six male and six female pups, where possible, on postnatal day (PND1). Dams and offspring were weighed every 3 days during lactation. All rats were weaned onto standard chow diet and housed three to four per cage. At weaning (3 weeks) and 14 weeks of age, offspring were anaesthetised by intraperitoneal injection of ketamine/xylazine and decapitated prior to tissue collection; dams were euthanised at weaning. Matched liver samples from eight chow and eight Caf dams and a male and a female sibling of each age were collected and snap frozen (48 in total). At 5 weeks old, all rats underwent a whole-body composition analysis by nuclear magnetic resonance imaging (EchoMRI), assessing their fat mass. All animal procedures were approved by the Animal Care and Ethics Committee of UNSW Sydney (#19/74A).

### Liver RNA-seq

Livers from dams and their respective offspring (at weaning and 14 weeks of age) underwent RNA-seq analysis. RNA was extracted from approximately 10 mg of ground liver tissue with a Qiagen RNeasy Plus Mini kit according to the manufacturer's instructions, supplemented with 1 M dithiothreitol. Total RNA underwent depletion of rRNA and library preparation using the Illumina Stranded Total RNA Prep Ligation kit with Ribo-Zero Plus at the Ramaciotti Centre for Genomics (UNSW).

All RNA-seq libraries (each group *n*=8) were sequenced using an Illumina NovaSeq to produce 150 nt paired-end reads for each sample. Read integrity and quality were confirmed using FastQC (v0.11.8) (Andrews, 2015). The reads were then mapped to the Ensembl *Rattus norvegicus* genome (Rattus_norvegicus.mRatBN7.2.dna). Mapping was performed with Subread (v 1.6.3) (Liao et al., 2013). The featureCounts function of Subread was used to generate counts of reads uniquely mapped to annotated genes using the Rattus_norvegicus.mRatBN7.2.105.gtf file.

DEG analysis was performed using the Bioconductor packages edgeR (v 3.38.4) (Robinson et al., 2010) and voom (limma) (Ritchie et al., 2015), using raw count data as input with normalisation performed using the Trimmed Mean of M-values method. Lowly expressed genes (cpm<0.5 in at least six samples) were filtered out, leaving 14,559 Ensembl IDs (13,192 genes with a unique HGNC symbol) for analysis. Differential expression was performed using limma (voom). Three samples (one male and one female weaner offspring from chow-fed mothers and one male adult offspring from a chow-fed mother) were excluded due to sample identity errors during sample processing, leaving *n*=7; all other groups had *n*=8. The functions lmFit (robust method), eBayes and treat were used to identify DEGs across different groupings with at least a log2(1.1) fold change. Significantly DEGs were taken as having an adjusted *P*<0.05. R plotting functions, including plotMDS (limma) and pheatmap R package, were used to prepare plots. The kegga function (limma) was used to perform KEGG pathway analysis.

### Liver RRBS of liver DNA at weaning

Four groups of rats were chosen for RRBS: 3-week-old female and male offspring from chow- or Caf-fed mothers. Five individual rats from separate mothers were selected per sex per group. DNA was extracted from 20-30 mg of ground liver with a Qiagen DNeasy Blood & Tissue Kit and eluted in AE buffer (10 mM Tris-HCl, 0.5 mM EDTA). 0.5 μg of DNA was sent to the Ramaciotti Centre for Genomics at UNSW Sydney, Australia, and underwent Zymo-Seq RRBS library preparation and Illumina NovaSeq 6000 SP 2×50 bp XP at 30 M read pairs per sample. Two female samples, one from each group, failed due to low 260:230 ratios and were excluded from analyses. Sequence analyses were performed on the Galaxy (2022) Australia Bioinformatics Platform (https://usegalaxy.org.au/). Sequence files were aligned to the rn7 rat genome with BWA-meth. Per-base CpG methylation metrics were extracted with MethylDackel, with the output limited to CpG sites with a minimum of five reads in coverage; the output is presented as CpG methylation fractions. DMRs between the groups were identified using Metilene, with a minimum of 10 CpGs per DMR and a minimum of 10% methylation difference between groups. DMRs were visualised, and nearby genes and orthologous genome regions were identified with the UCSC Genome Browser (https://genome.ucsc.edu/ with Table Browser and LiftOver tools) or Ensembl BioMart (https://asia.ensembl.org/info/data/biomart/index.html). The gene ontology of the DMR-proximal genes was performed with the Database for Annotation, Visualization and Integrated Discovery (DAVID) (https://davidbioinformatics.nih.gov/).

Colocalisation of DMRs and genome elements was performed on the Galaxy (2022) Australia Bioinformatics Platform (https://usegalaxy.org.au/). Rn7 bed files with gene locations [UCSC Main on Rat: ncbiRefSeq (genome)], CGIs [UCSC Main on Rat: cpgIslandExt (genome)], and RepeatMasker [UCSC Main on Rat: rmsk (genome)] coordinates were imported into Galaxy with the Get Data function, UCSC Main Table Browser. Rat liver-primed and active enhancer regions were previously identified by Roller et al. (2021), and rn7 genome coordinates were imported from the EBI FTP site (ftp.ebi.ac.uk/pub/software/vertebrategenomics/FOG29/mergeRegRegions). Bed files were curated with Filter (Galaxy Version 1.1.1) and Advanced Cut (Galaxy Version 9.5+galaxy2) before colocalisation, with DMRs being determined with Bedtools WindowBed (Galaxy Version 2.31.1).

### Ethical approval

Ethical approval for the study was received from the UNSW Animal Ethics Committee (#19/74A).

## Supplementary Material



10.1242/biolopen.062573_sup1 Supplementary information

Dataset 1.
