# Supplementary material for: Maternal obesity in rats results in male-specific increases in genome-wide DNA methylation in postnatal offspring liver
Source: Biol Open. 2026 Sep 1;15(9):bio062573. doi: 10.1242/bio.062573 (PMC13580582; doi:10.1242/bio.062573)
Supplement: Supplementary file 1 [file biolopen-15-062573-s1.pdf]

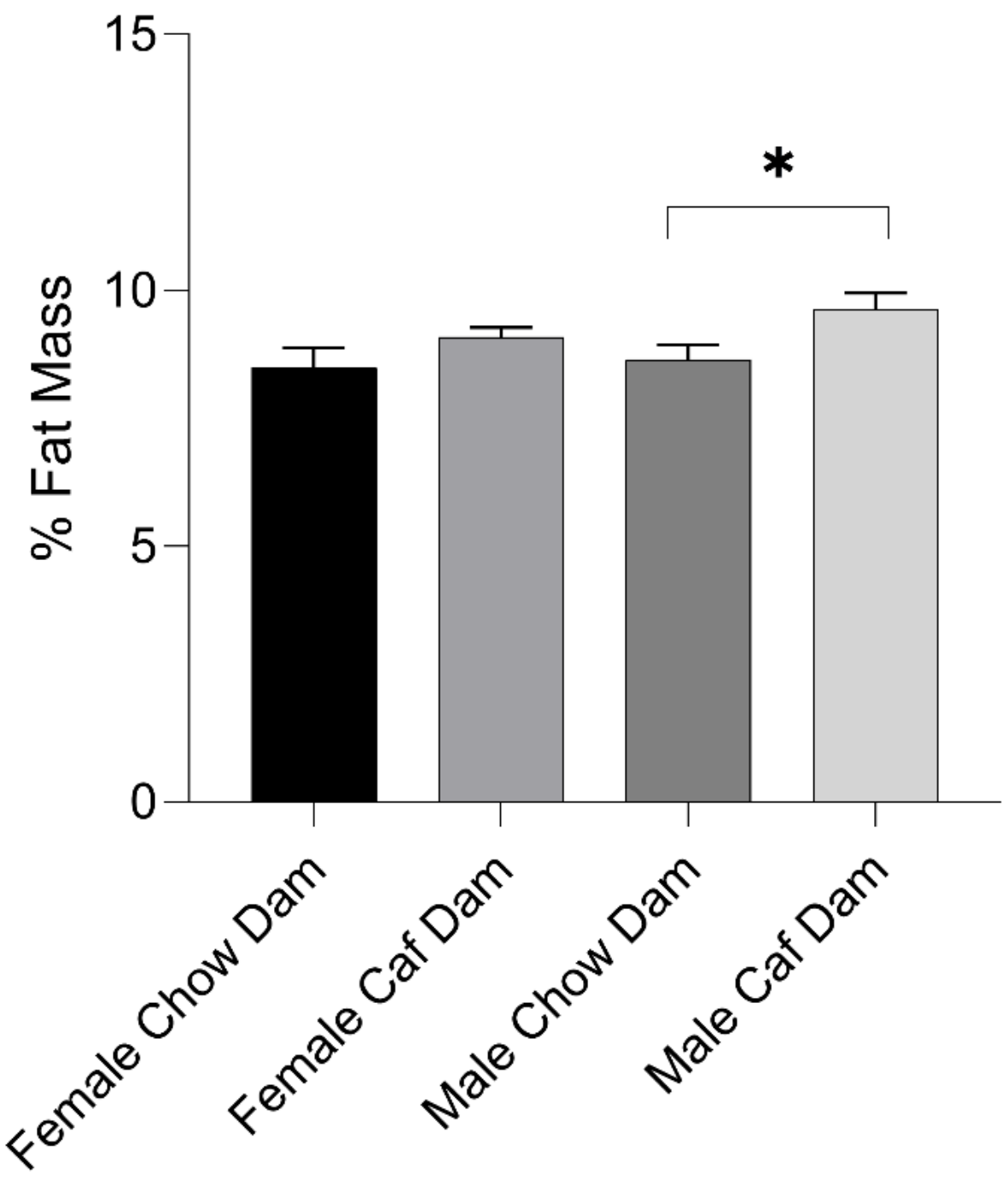

Fig. S1. Percentage of body weight composed of fat mass in weaner offspring measured by echoMRI. Fat mass was significantly increased in male offspring of Caf-fed dams compared to male offspring of Chow-fed dams. N=8 per group, analysed by 2-way ANOVA. P=0.02 main effect of maternal diet, multiple group comparison P=0.04 difference in male offspring presented, mean ± SEM.

Biology Open • Supplementary information

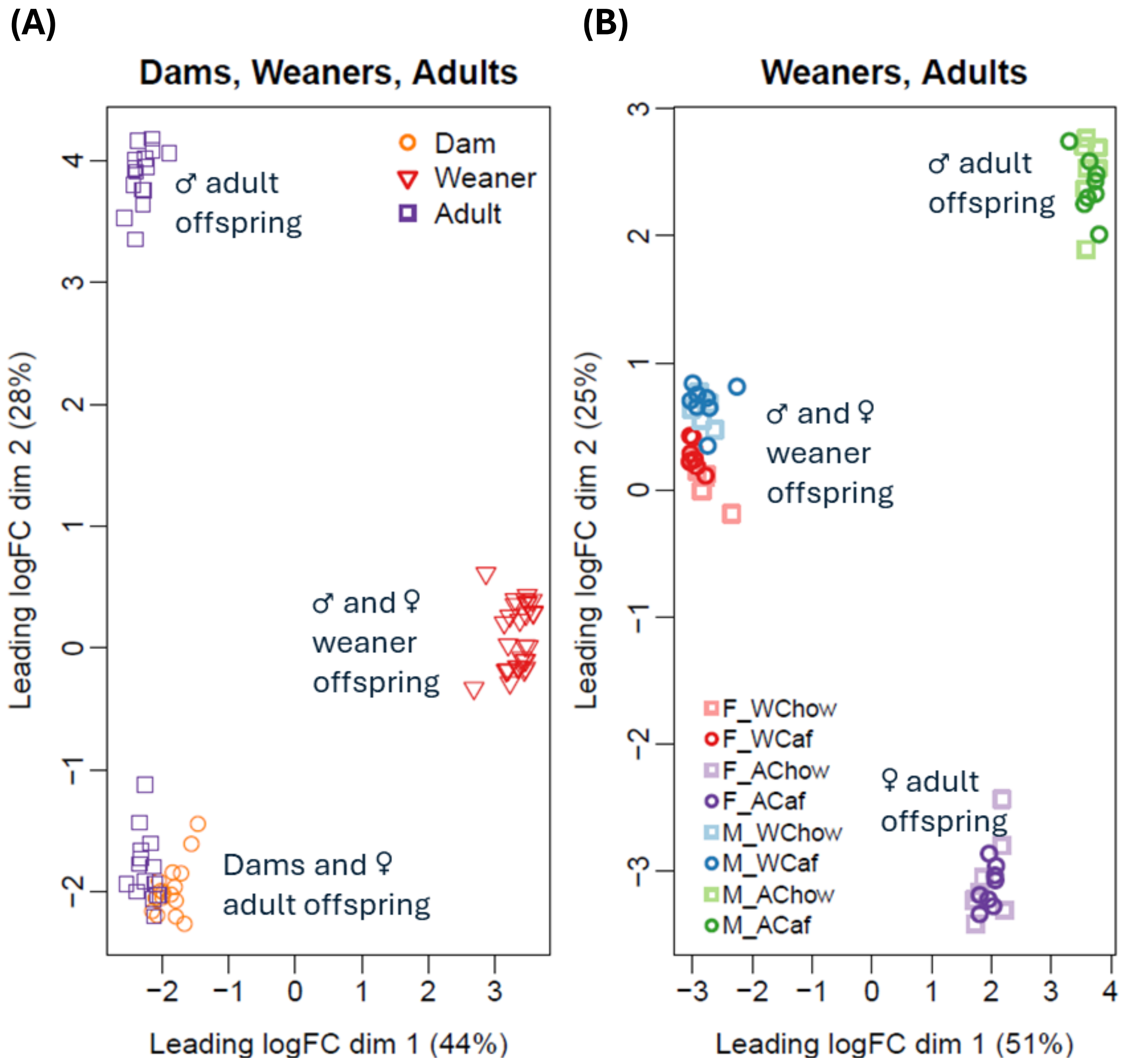

Fig. S2. RNA-seq analysis: Multidimensional scaling (MDS) plots showing (A) overall age and sex effects including dams and offspring and (B) maternal diet and sex effects in male and female offspring at weaner and adult ages.

WChow weaner from Chow fed dam; WCaf weaner from Caf fed dam; AChow 14 week offspring from Chow fed dam; ACaf 14 week offspring from Caf fed dam. F female, M male. See colour code insets in figures describing subgroups.

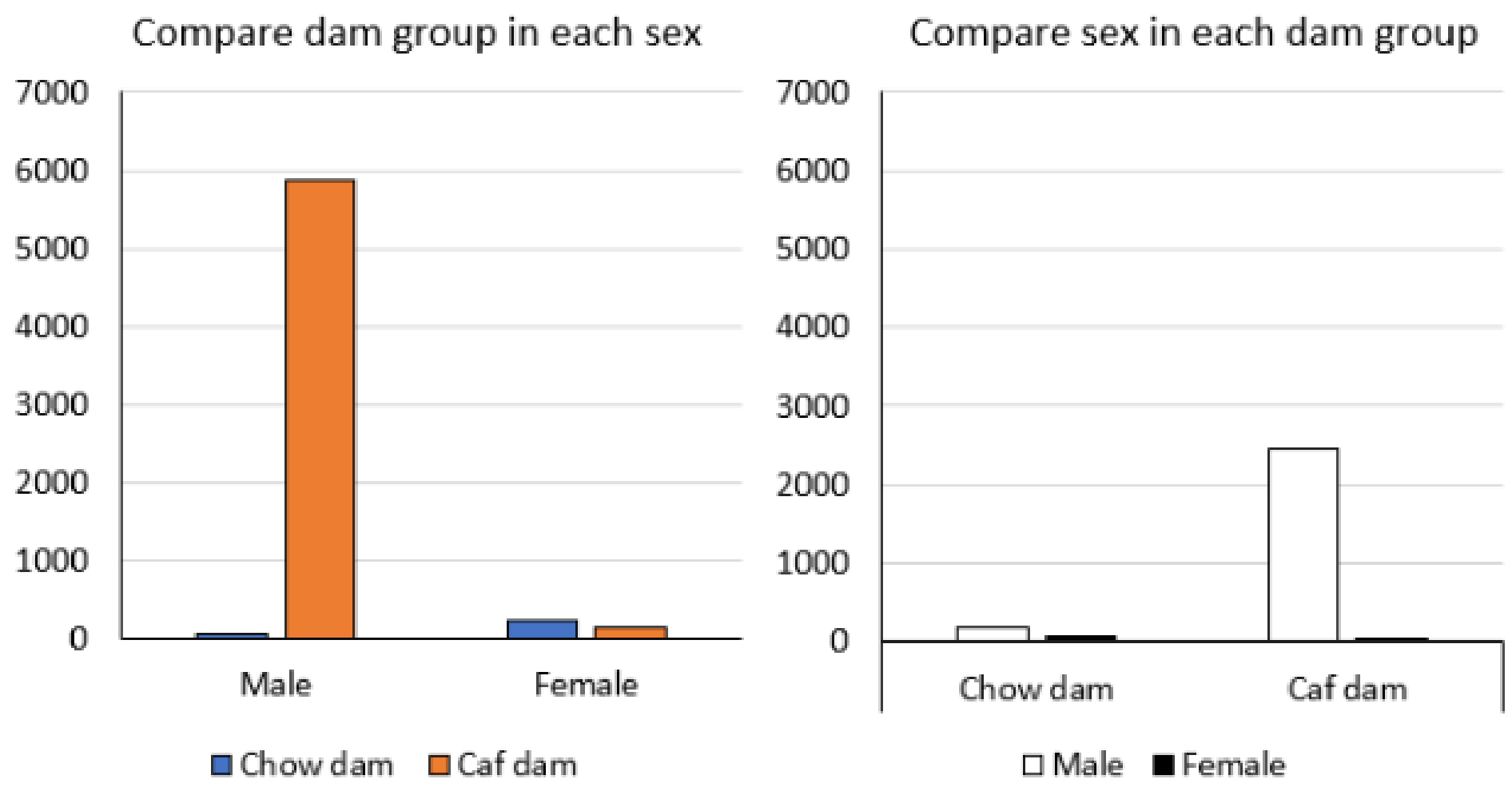

Fig. S3. Comparison of number of DMRs with higher methylation in each offspring group comparison.

P<0.05 DMRs from four 2-group comparisons of RRBS data for offspring at weaning. For example, the group furthest on the left compares methylation in male offspring from chow-fed dams versus male offspring from cafeteria-diet fed dams. This identified 45 DMRS with significantly higher methylation in the Chow dam group and 5879 DMRS with significantly higher methylation in the Caf dam group.

(A)

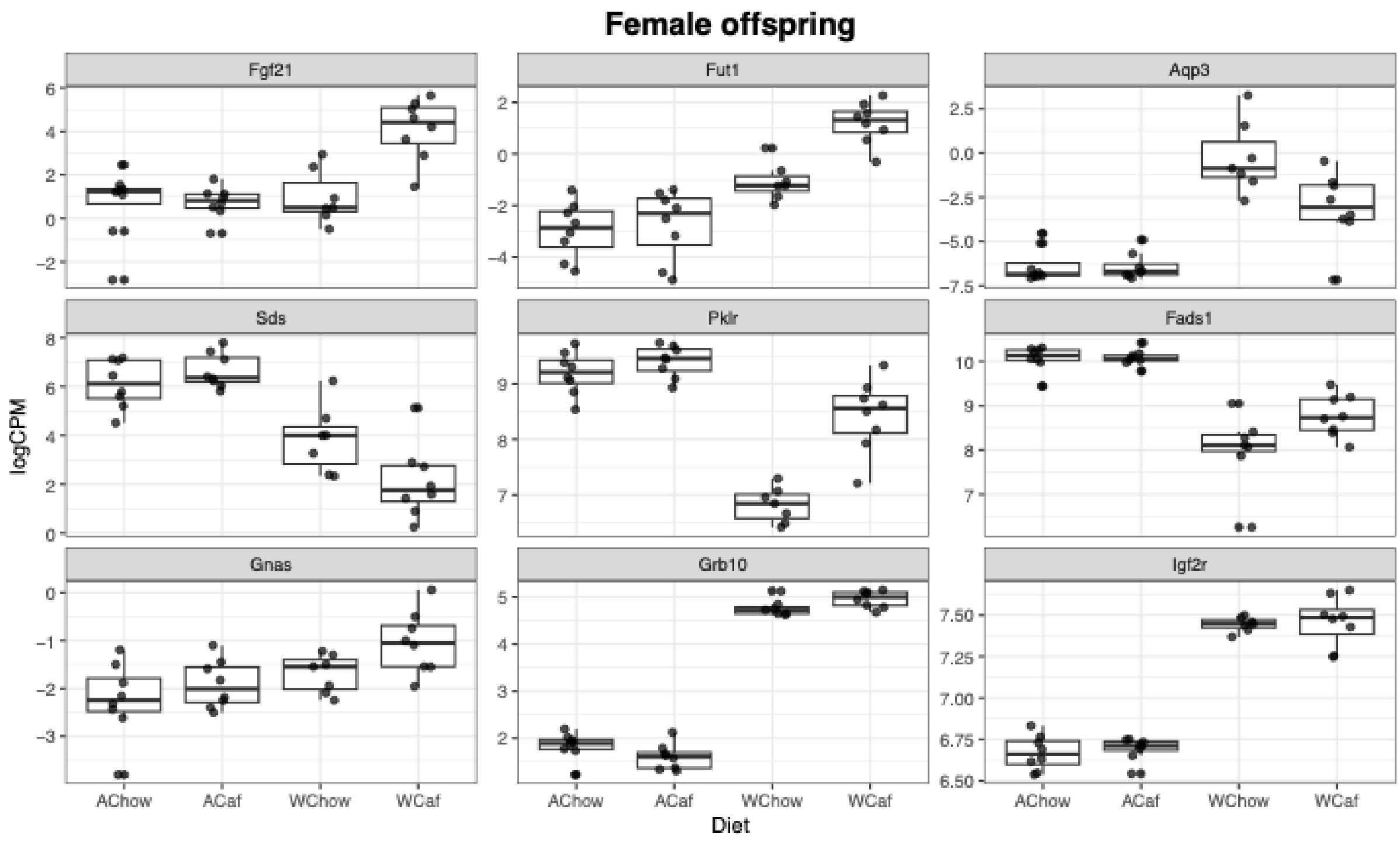

(B)

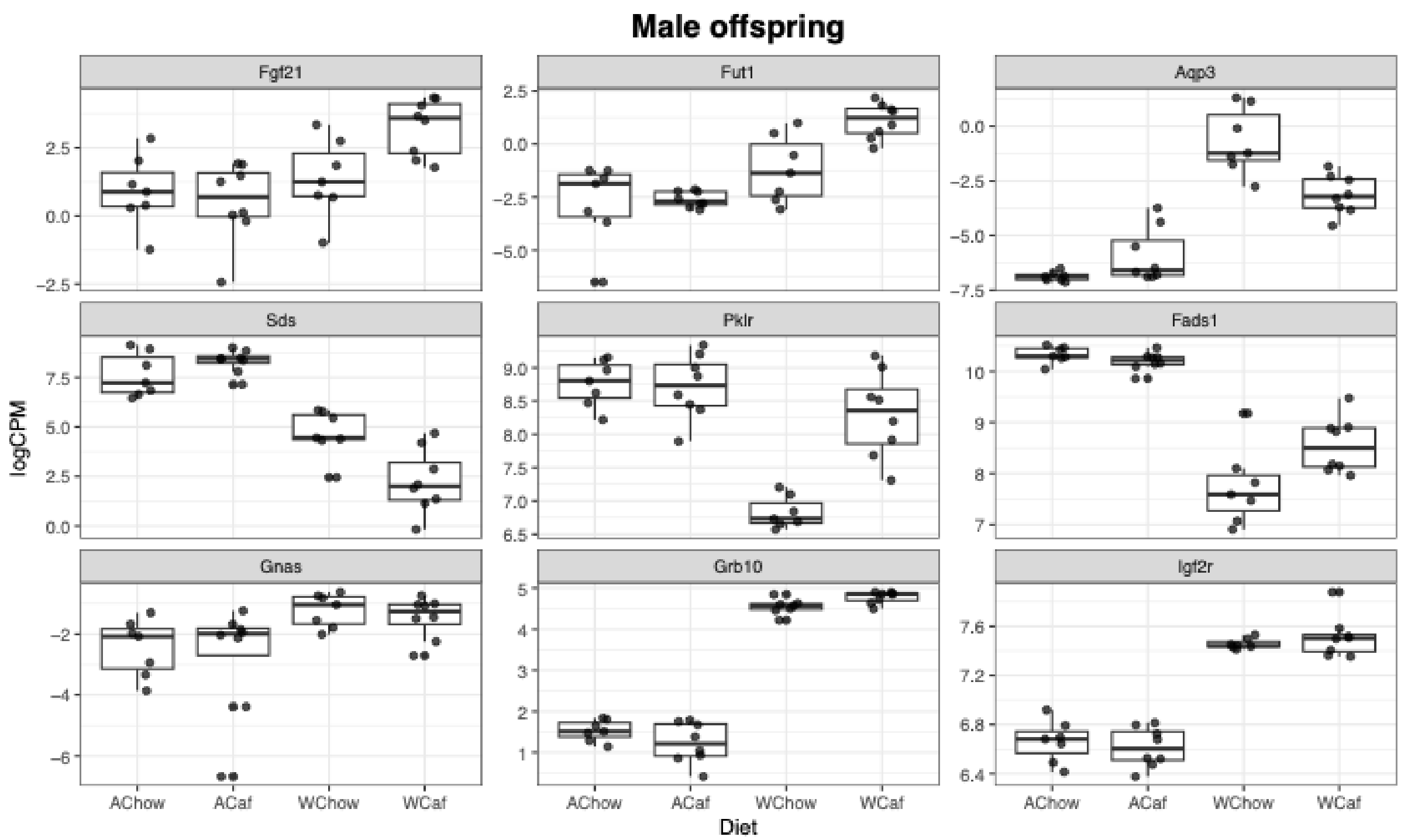

Biology Open • Supplementary information

Fig. S4. Expression of selected significant DEGs in female and male offspring livers. Boxplots show normalized RNA-seq expression (logCPM) for genes in female (A) and male (B) offspring at weaning and adulthood from Chow-fed (Chow) and cafeteria diet-fed (Caf) dams. All genes were DEGs within 10Kb from a DMRs in male weaners. Genes were selected to illustrate examples discussed in the comparison of differential gene expression and nearby differentially methylated regions (DMRs), including genes exhibiting sex-differences in methylation but similar gene expression (e.g. *Fgf21*, *Fut1*, *Aqp3* and *Sds*) and selected imprinted genes (*Gnas, Grb10, Igf2r*). Individual points represent biological replicates and boxplots show the median and interquartile range.

Dataset 1.

Available for download at
https://journals.biologists.com/bio/article-lookup/doi/10.1242/bio.062573#supplementary-data

Biology Open • Supplementary information

Table S1. Genomic elements located at the liver DMRs of each comparison at weaning. Number of genomic elements that overlapped with DMR regions listed with % of total DMRs in brackets. Percentages can be above 100 as an individual DMR can be present at multiple genomic elements (e.g. multiple TSS at genes with multiple or shared promoters). M, male; F, Female; Ch, offspring of chow-fed mother; Caf, offspring of cafeteria-diet mother, CGI, CpG island; TSS, transcriptional start site; W weaner.

	**Total DMRs 5924**	**Total DMRs 363**	**Total DMRs 913**	**Total DMRs 2754**
	MW Ch vs Caf	FW Ch vs Caf	FW Ch vs MW Ch	FW Caf vs MW Caf
**+/-1Kb of gene TSS**	2113 (35.7)	188 (51.8)	2277 (249.4)	1997 (72.5)
**+/-1Kb of CGI**	917 (15.5)	141 (38.8)	690 (75.6)	741 (26.9)
**Repetitive DNA**	2056 (34.7)	103 (28.4)	208 (22.8)	787 (28.6)
**+/-1Kb of active enhancers**	345 (5.8)	16 (4.4)	21 (2.3)	177 (6.4)
**+/-1Kb of primed enhancers**	398 (6.7)	22 (6.1)	47 (5.1)	142 (5.2)

Biology Open • Supplementary information

Table S2. Correlation between differentially expressed genes (DEGs) and nearby differentially methylated regions (DMRs) suggests no association between regional methylation and expression levels. Negative values in DMR change indicates higher methylation in offspring from Caf-fed dams. Negative values in DEG change indicates lower expression in offspring from Caf-fed dams. Genes within groups in alphabetical order.

DEGs in female weaners with DMRs within 10kb		
**Gene**	**DMR change**	**Transcript change**
Aqp3	0.18	-2.18
Dcaf11	0.14	-0.4
Ddit4	0.11	1.06
Fgf21	0.17	3.46
Fut1	0.14	2.3
Hamp	-0.11	-9.22
Hspb8	0.1	-0.82
**Female weaner Pearson correlation coefficient 0.85**		

DEGs in male weaners with DMRs within 10kb		
**Gene**	**DMR change**	**Transcript change**
Aqp3	-0.32	-2.05
Cavin3	-0.17	1.03
Cbs	-0.17	-0.6
Dhtkd1	-0.12	-0.44
Eno1-ps20	-0.21	0.58
Fads1	-0.21	0.87
Fgf21	-0.12	2.04
Fut1	-0.12	2.43
Gjc2	-0.13	0.94
Gstm7	-0.11	2.08
Gstp3	-0.14	-0.97
Mogat3	-0.16	1.48
Mt-co1	-0.16	-0.46
Mt-nd1	-0.16	-0.51
Mt-nd5	-0.16	-0.47
Pc	-0.13	0.45
Pklr	-0.11	1.47
Plod3	-0.16	0.39
Pycr1	-0.19	1.18
Sds	-0.11	-2.61
Slc25a1	0.15	0.44
Slc2a5	-0.11	1.87
**Male weaner Pearson correlation coefficient 0.24**		

DEGs in male adults with DMRs within 10kb		
**Gene**	**DMR change**	**Transcript change**
Clstn1	-0.22	-0.68
Slc30a2	-0.16	-1.73
Togaram2	-0.17	0.86
Zbtb7c	-0.11	1.43
**Male adult Pearson correlation coefficient 0.51**		
