# Supplementary material for: Maternal obesity in rats results in male-specific increases in genome-wide DNA methylation in postnatal offspring liver
Source: Biol Open. 2026 Sep 1;15(9):bio062573. doi: 10.1242/bio.062573 (PMC13580582; doi:10.1242/bio.062573)
Supplement: Supplementary file 2 [file biolopen-15-062573_review_history.pdf]

Maternal obesity in rats results in male-specific increases in genome-wide DNA methylation in postnatal offspring liver

Susan M. Corley, Kyoko Hasebe, Aynaz Tajaddini, Michael D. Kendig, Margaret J. Morris and Neil A. Youngson
10.1242/bio.062573

Editor: Tristan Rodríguez

Review timeline

Original submission:	16 March 2026
Editorial decision:	20 March 2026
First revision received:	22 July 2026
Accepted:	23 July 2026

Original submission

First decision letter

MS ID#: bio.062573

MS Title: Maternal obesity in rats results in male-specific increases in genome-wide DNA methylation in postnatal offspring liver

Authors: Neil A. Youngson; Susan M. Corley; Kyoko Hasebe; Aynaz Tajaddini; Michael D. Kendig; Margaret J. Morris

I have now reached a decision on the above manuscript.

The reviewer reports are shown at the bottom of this email.

As you will see, the reviewers raised a number of substantial criticisms that prevent me from accepting the paper at this stage.

They suggest, however, that a revised version might prove acceptable, if you can address their concerns. If you think that you can deal satisfactorily with the criticisms on revision, I would be pleased to see a revised manuscript. We would then return it to the reviewers.

At this stage, we also ask you to ensure your manuscript complies with our formatting guidelines. Provided you are able to fully address the referees' comments, we are positive about publication of your paper (we accept over 95% of revision submissions) and therefore hope you won't mind any extra work involved in reformatting your manuscript at this point.

Please upload both a 'clean' version of your Word file, along with a highlighted version clearly showing where you have made changes in the revised manuscript. Please avoid using 'Track changes' in Word files as these are lost in PDF conversion.

I should be grateful if you would also provide a point-by-point response detailing how you have dealt with the points raised by the reviewers in the 'Response to Reviewers' box. Please attend to all of the reviewers' comments. If you do not agree with any of their criticisms or suggestions please explain clearly why this is so.

© 2026. Published by The Company of Biologists under the terms of the Creative Commons Attribution License (https://creativecommons.org/licenses/by/4.0/).

Reviewer 1

Comments for the author

Youngson et al. investigate whether male-specific peripubertal DNA demethylation observed in mice is conserved in rats and how maternal obesity influences offspring liver epigenetics and gene expression. Using a cafeteria diet model, the authors show that maternal obesity alters offspring growth and adiposity in a sex-specific manner. Contrary to findings in mice, no evidence of male-specific DNA demethylation was observed in rat offspring. However, maternal diet had a pronounced impact on DNA methylation, particularly in males, where the majority of differentially methylated regions exhibited higher methylation in offspring from obese dams. Despite these epigenetic changes, integration with transcriptomic data suggests that DNA methylation does not account for most sex-specific gene expression differences at weaning. Overall, the study highlights species differences and the strong environmental sensitivity of male liver methylation during early development. The authors need to clarify several statements in the manuscript before acceptance, and detailed comments are provided below.

Major concerns:

1) The initial liver RNA-seq experiments exploring transcriptional response by maternal diet present the use of log2-transformed CPM values. This normalization approach is suboptimal and may introduce bias into the analysis. I strongly recommend that the authors re-analyze the data using TPM or another appropriate normalization method.

2) The adult males DEGs have shown a lipid phenotype; this needs to be further validated from the qPCR or metabolic assays from the same cohort in comparison to chow-fed dams and caf-fed dams.

3) Although the authors have made the overlap and intersection of DMRs and DEGs, I highly recommend a Spearman or Pearson correlation coefficient (rho) between methylation percentages of DMRs and normalized expression values (TPM) to further support your claim.

4) Authors mentioned in the abstract that "strong maternal diet effects in male offspring were observed", they need to clarify where the changes observed in weaners or adult males in comparison to caf-fed vs. chow-fed maternal diets? I do not find strong supporting evidence for this claim unless addressing Comment 2 from the major concerns.

Minor concerns:

1) In line 260, the authors mentioned 37 genes with differential expression from adult males. Is it the total of 36 DEGs from this group?

2) What is the rationale for choosing a cafeteria diet over a readymade high-fat diet for rats?

3) How is normalization performed for RNA-seq analysis?

4) In the Figure 1 legends, authors should mention where the DEGs are coming from? Are these DEGs common in maternal obesity in both male and female weaner offspring?

5) I request the authors to submit the RNA-seq and bisulphite-seq raw data or the sequence files in GEO database.

Reviewer 2

Comments for the author

Youngson et al. ask whether the male-specific postnatal liver demethylation program described in mice born to obese mothers is conserved in rats. The study combines RRBS and RNA-seq and reports

© 2026. Published by The Company of Biologists under the terms of the Creative Commons Attribution License (https://creativecommons.org/licenses/by/4.0/).

a striking male-specific increase in DNA methylation at weaning in offspring of Caf-fed (obese) dams.

The manuscript is interesting, but there are some points that need to be addressed.

1. There is an issue in how the findings are described. The abstract and framing refer to "increased genome-wide demethylation," whereas the reported data indicate that the vast majority of DMRs in males are hypermethylated. As written, this is logically inconsistent. It would be useful to clarify whether the authors intend to describe a reduction or failure of normal demethylation, rather than increased demethylation per se, and to ensure consistent terminology throughout the manuscript. Basically, the direction of methylation needs to be clearly stated.

2. All methylation analyses are conducted at a single developmental stage (3 weeks). Given that the mouse demethylation process spans a broader postnatal window, the absence of a comparable pattern in the rat may reflect developmental timing rather than disruption. This possibility should be stated.

3. The overlap between DMRs and DEGs is limited, particularly in males. While this is acknowledged, the manuscript still implies functional consequences that are not strongly supported by the data. Additionally, the first time DMR is mentioned, it should be defined.

4. KEGG pathway enrichment results are presented; however, none of the identified pathways remained significant after multiple testing correction. Despite this, these pathways are discussed in a biological context. These findings could be suggestive, as there is not robust support for mechanism.

Reviewer's Responses to Questions

Experimental quality

Does each figure have the proper controls?
If 'No', please indicate reasons in Comments for Author box below.

Reviewer #1:
- Yes

Reviewer #2:
- Yes

Were the data analyzed using appropriate statistical tests?
If 'No', please indicate reasons in Comments for Author box below.
Reviewer #1:
- No

Reviewer #2:
- No

Reproducibility

Were experiments performed using adequate number of biological replicates?
If 'No', please indicate reasons in Comments for Author box below.
Reviewer #1:
- Yes

Reviewer #2:
- Yes

Does the methods section provide sufficient detail to permit reproducibility?
If 'No', please indicate reasons in Comments for Author box below.
Reviewer #1:
- Yes

© 2026. Published by The Company of Biologists under the terms of the Creative Commons Attribution License (https://creativecommons.org/licenses/by/4.0/).

Reviewer #2:

- Yes

Completeness
Are the manuscript's conclusions supported by the data?
If 'No', please indicate reasons in Comments for Author box below.
Reviewer #1:

- No

Reviewer #2:

- No

Scholarship

Do the authors cite and discuss the merits of data that would argue for and against their conclusion?
If 'No', please indicate reasons in Comments for Author box below.
Reviewer #1:

- Yes

Reviewer #2:

- Yes

Does the manuscript title & abstract accurately reflect the contents of the manuscript, without hyperbole?
If 'No', please indicate reasons in Comments for Author box below.
Reviewer #1:

- Yes

Reviewer #2:

- No

First revision

Author response to reviewers' comments

17th July 2026

Dear Professor Rodriguez,

Thank you for your patience in extending our manuscript review deadline and for finding some excellent reviewers for the manuscript. All of their suggestions were insightful and/or appropriate, and we feel the study is now much improved. We have reviewed the manuscript according to their suggestions as is detailed in a point-by-point response below. In the revised manuscript the changes are indicated in red text.

Comments from the Reviewers:

Reviewer 1*: Youngson et al. investigate whether male-specific peripubertal DNA demethylation observed in mice is conserved in rats and how maternal obesity influences offspring liver epigenetics and gene expression. Using a cafeteria diet model, the authors show that maternal obesity alters offspring growth and adiposity in a sex-specific manner. Contrary to findings in mice, no evidence of male-specific DNA demethylation was observed in rat offspring. However, maternal diet had a pronounced impact on DNA methylation, particularly in males, where the majority of differentially methylated regions exhibited higher methylation in offspring from obese dams. Despite these epigenetic changes, integration with transcriptomic data suggests that DNA methylation does not account for most sex-specific gene expression differences at weaning. Overall, the study highlights species differences and the strong environmental sensitivity of male liver methylation during early development. The authors need to clarify several statements in the manuscript before acceptance, and detailed comments are provided below.*

© 2026. Published by The Company of Biologists under the terms of the Creative Commons Attribution License (https://creativecommons.org/licenses/by/4.0/).

Major concerns:

1) The initial liver RNA-seq experiments exploring transcriptional response by maternal diet present the use of log2-transformed CPM values. This normalization approach is suboptimal and may introduce bias into the analysis. I strongly recommend that the authors re-analyze the data using TPM or another appropriate normalization method.

Response: We are grateful for the reviewer's understanding of the value of the study and their valuable comments on areas to be improved.

However, we do not agree with this suggestion. Differential expression (DE) analysis was performed using edgeR (Robinson, McCarthy, Smyth [2010]) DOI: 10.1093/bioinformatics/btp616 (NB > 24,000 citations) which requires raw count data, not normalised data. edgeR models the RNA count data using the negative binomial distribution, which accounts for the mean-variance relationship inherent in such data. As part of this framework, edgeR performs normalisation using the Trimmed Mean of M-values (TMM) method (Robinson, Oshlack [2010] DOI: 10.1186/gb-2010-11-3-r25).

TPM values would not be appropriate input for edgeR (or DESeq2) as they are not integer counts and therefore violate the assumptions of the negative binomial model.

TPM values are often used for comparison of gene expression *within* a sample, but this is not required for comparison of gene expression *between* different samples, as is our study design.

We have added reference to the TMM method into the paragraph dealing the RNA-seq analysis to ensure that our approach is not misunderstood.

Lines 444-446 "Differential gene expression (DEG) analysis was performed using the Bioconductor packages, edgeR (v 3.38.4)(Robinson et al., 2010) and voom (limma)(Ritchie et al., 2015), using raw count data as input with normalisation performed using the Trimmed Mean of M-values (TMM) method."

2) The adult males DEGs have shown a lipid phenotype; this needs to be further validated from the qPCR or metabolic assays from the same cohort in comparison to chow-fed dams and caf-fed dams.

Response: We think the reviewer means male weaners rather than male adults having a lipid phenotype. As stated on lines 179-181 "KEGG analysis revealed no enriched pathways in male or female adult offspring of obese mothers." However, lines 167-170 describes the significant enrichment of Fatty acid metabolism in the DEGs with higher levels in Caf diet group in males **at weaning**. "The few differences between the male and female weaners' responses to maternal Caf diet were the significant enrichment of Fatty acid metabolism in the DEGs with higher levels in Caf diet group in males,".

We have now added **Supplementary Figure 1** to describe the increase in body fat in male, but not female weaners. Therefore, the evidence for lipid phenotypes due to maternal obesity now includes female weaner body weight, liver score (i.e. adiposity) and liver fat metabolism genes (e.g. *Scd*, *Fads1*, *Fgf21*) whereas the male weaner evidence comes from liver score, whole body lipid content and liver fat metabolism genes (e.g. *Scd*, *Fads1*, *Fgf21*). The gene expression patterns of *Fads1* and *Fgf21* genes is also displayed in **Supplementary Figure 4** which was primarily generated in response to point 3, below.

© 2026. Published by The Company of Biologists under the terms of the Creative Commons Attribution License (https://creativecommons.org/licenses/by/4.0/).

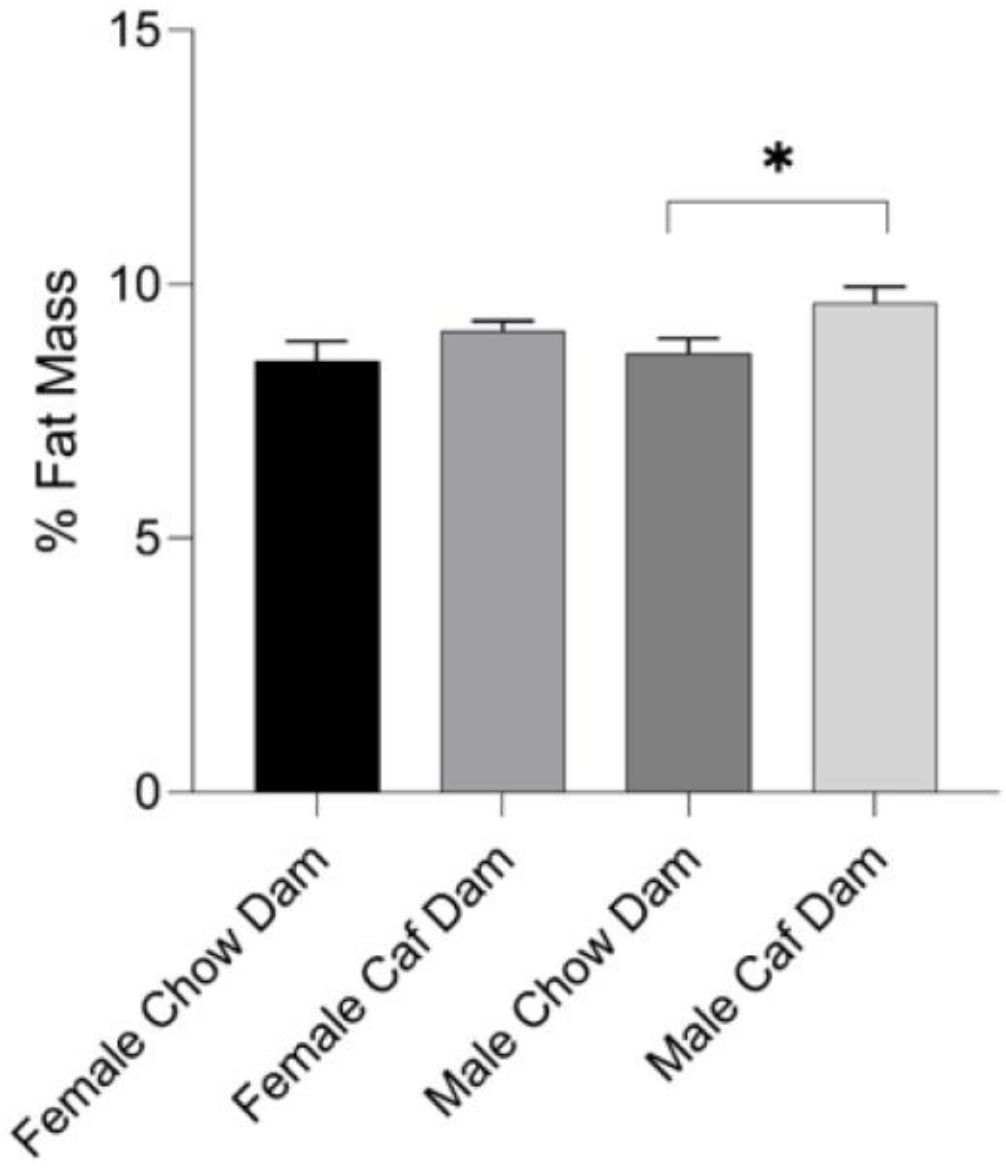

Supplementary Figure 1. Percentage of body weight composed of fat mass in weaner offspring measured by echoMRI. Fat mass was significantly increased in male offspring of Caf-fed dams compared to male offspring of Chow-fed dams. N=8 per group, analysed by 2-way ANOVA. P=0.02 main effect of maternal diet, multiple group comparison P=0.04 difference in male offspring presented, mean ± SEM.

New text in the manuscript:

Methods section lines 427-429, "At 5 weeks old, all rats underwent a whole-body composition analysis by nuclear magnetic resonance imaging (EchoMRI) assessing their fat mass."

Results section lines 130-132, "At weaning, whole body fat mass was significantly increased in male offspring of Caf-fed dams compared to male offspring of Chow-fed dams. A similar but not significant trend was seen in female siblings (Supplementary Figure 1)."

3) Although the authors have made the overlap and intersection of DMRs and DEGs, I highly recommend a Spearman or Pearson correlation coefficient (rho) between methylation percentages of DMRs and normalized expression values (TPM) to further support your claim.

Response: We think that this comment is meant for the section 'DMRs at weaning do not underlie the majority of transcriptional differences in weaner and adult offspring livers'

We have produced a supplemental correlation coefficient table for the significant DEGs that have a DMR within 10Kb (7 in female weaner, 22 in male weaners, 4 in adult males). This is now included in the manuscript in lines 266-278 (**Supplementary Table 2**). We have also included a new **Supplementary Figure 4** showing gene expression in 9 of the genes referred to in this section.

"Pearson correlation was undertaken to examine the relationship between methylation and transcription at DEGs with DMRs within 10kb (Supplementary Table 2). This analysis does identify a few genes with strong associations of high methylation/low expression or vice versa, such as Hamp and Fgf21 in female weaners. The Pearson correlation coefficient for these 7 genes is strong at 0.85, but drops to a negligible 0.005 when Hamp is excluded, revealing that it is driving the group correlation. However, for males in which 99% of DMRs have higher methylation in offspring of Caf-dams, the 22 genes with nearby DMRs had only a weak 0.24 Pearson correlation coefficient. Only 4 DEGs in adult males had a nearby DMR, and these had a moderate Pearson correlation coefficient of 0.5. Therefore, the genome-wide DNA methylation increases in weaner male offspring of Caf-fed dams compared to chow-fed dams appears to not have caused a general decrease in gene expression.

© 2026. Published by The Company of Biologists under the terms of the Creative Commons Attribution License (https://creativecommons.org/licenses/by/4.0/).

The disconnect between methylation and expression is also illustrated in Supplementary Figure 4 which shows similar expression patterns of genes in males and females genes, in spite of significant differences in nearby DMRs at weaning.“

**DEGs in female weaners with DMRs within 10kb**		
**Gene**	**DMR change**	**Transcript change**
Aqp3	0.18	-2.18
Dcaf11	0.14	-0.4
Ddit4	0.11	1.06
Fgf21	0.17	3.46
Fut1	0.14	2.3
Hamp	-0.11	-9.22
Hspb8	0.1	-0.82
**Female weaner Pearson correlation coefficient 0.85**		

**DEGs in male weaners with DMRs within 10kb**		
**Gene**	**DMR change**	**Transcript change**
Aqp3	-0.32	-2.05
Cavin3	-0.17	1.03
Cbs	-0.17	-0.6
Dhtkd1	-0.12	-0.44
Eno1-ps20	-0.21	0.58
Fads1	-0.21	0.87
Fgf21	-0.12	2.04
Fut1	-0.12	2.43
Gjc2	-0.13	0.94
Gstm7	-0.11	2.08
Gstp3	-0.14	-0.97
Mogat3	-0.16	1.48
Mt-co1	-0.16	-0.46
Mt-nd1	-0.16	-0.51
Mt-nd5	-0.16	-0.47
Pc	-0.13	0.45
Pklr	-0.11	1.47
Plod3	-0.16	0.39
Pycr1	-0.19	1.18
Sds	-0.11	-2.61
Slc25a1	0.15	0.44
Slc2a5	-0.11	1.87
**Male weaner Pearson correlation coefficient 0.24**		

**DEGs in male adults with DMRs within 10kb**		
**Gene**	**DMR change**	**Transcript change**
Clstn1	-0.22	-0.68
Slc30a2	-0.16	-1.73
Togaram2	-0.17	0.86
Zbtb7c	-0.11	1.43
**Male adult Pearson correlation coefficient 0.51**		

© 2026. Published by The Company of Biologists under the terms of the Creative Commons Attribution License (https://creativecommons.org/licenses/by/4.0/).

Supplementary Table 2. Correlation between differentially expressed genes (DEGs) and nearby differentially methylated regions (DMRs) suggests no association between regional methylation and expression levels. Negative values in DMR change indicates higher methylation in offspring from Caf-fed dams. Negative values in DEG change indicates lower expression in offspring from Caf-fed dams. Genes within groups in alphabetical order.

(A)

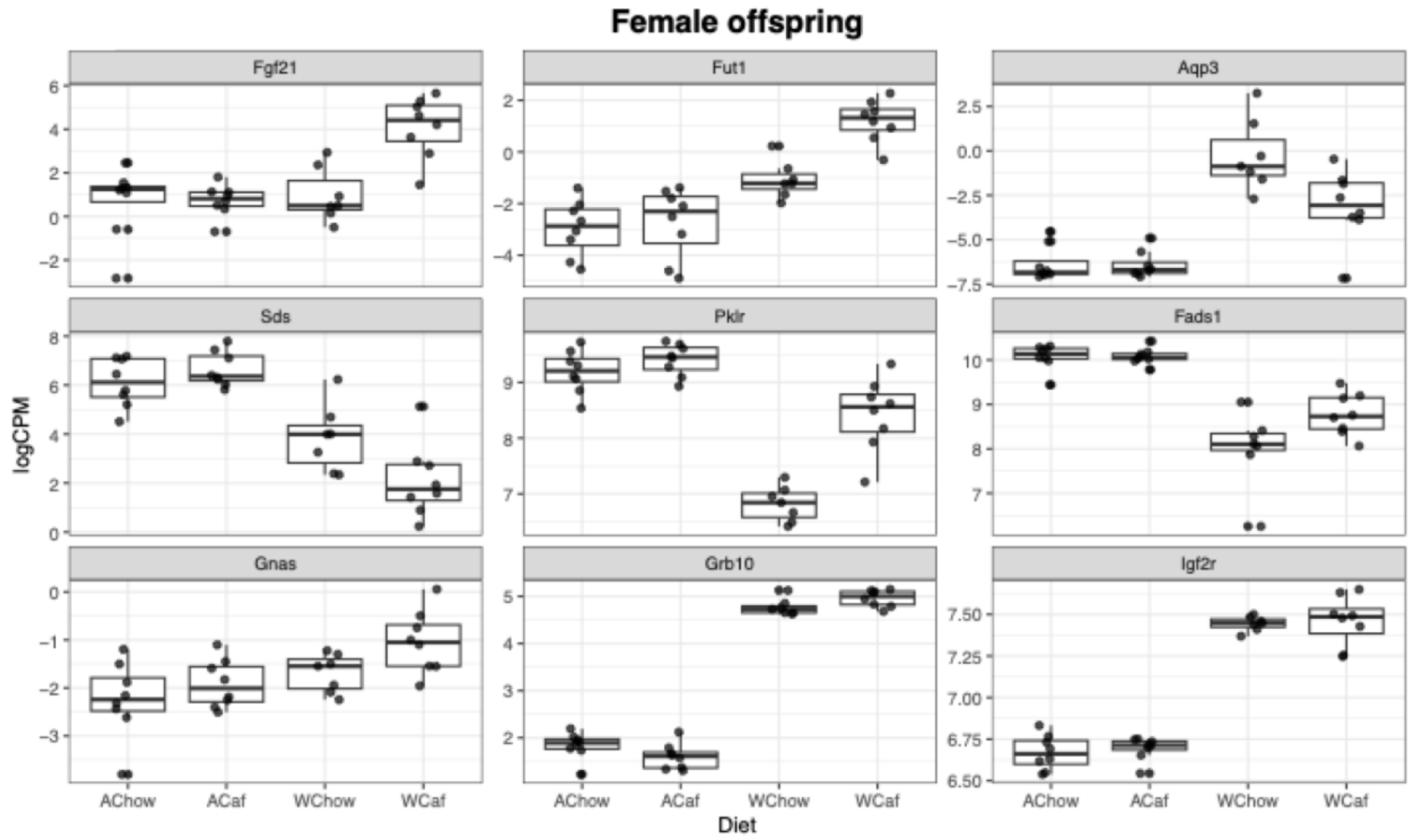

(B)

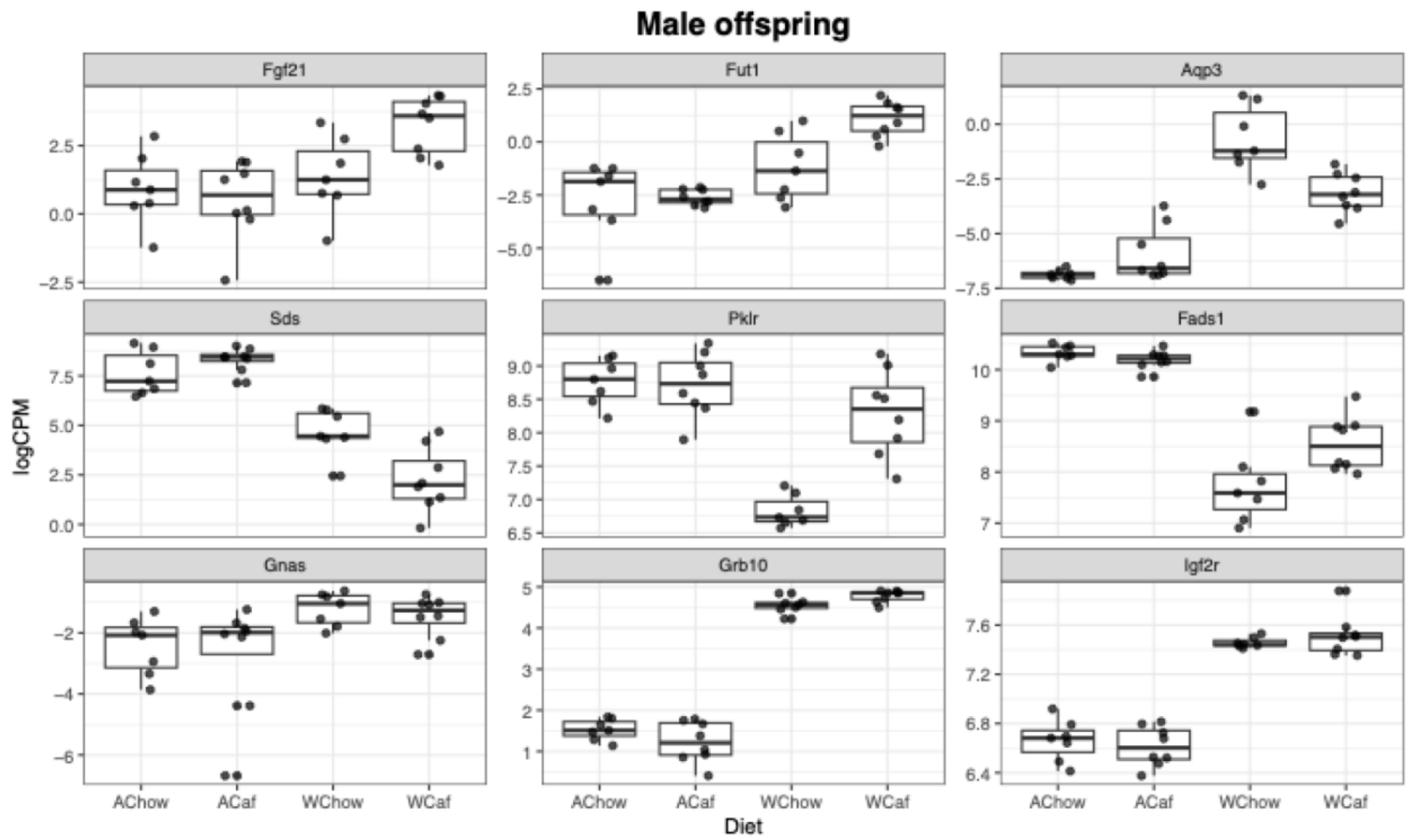

© 2026. Published by The Company of Biologists under the terms of the Creative Commons Attribution License (https://creativecommons.org/licenses/by/4.0/).

Supplementary Figure 4. Expression of selected significant DEGs in female and male offspring livers. Boxplots show normalized RNA-seq expression (logCPM) for genes in female (A) and male (B) offspring at weaning and adulthood from Chow-fed (Chow) and cafeteria diet-fed (Caf) dams. All genes were DEGs within 10Kb from a DMRs in male weaners. Genes were selected to illustrate examples discussed in the comparison of differential gene expression and nearby differentially methylated regions (DMRs), including genes exhibiting sex-differences in methylation but similar gene expression (e.g. *Fgf21*, *Fut1*, *Aqp3* and *Sds*) and selected imprinted genes (*Gnas, Grb10, Igf2r*). Individual points represent biological replicates and boxplots show the median and interquartile range.

4) Authors mentioned in the abstract that "strong maternal diet effects in male offspring were observed", they need to clarify where the changes observed in weaners or adult males in comparison to caf-fed vs. chow-fed maternal diets? I do not find strong supporting evidence for this claim unless addressing Comment 2 from the major concerns.

Response: We apologise for the lack of clarity in the abstract on this point. This comment was meant to apply to DNA methylation. Namely that 99% of DMRS were higher methylated in male offspring from Caf-fed mothers. We have amended the sentence to read “However, strong maternal diet effects in male weaner offspring methylation were observed.”

Minor concerns:

1) In line 260, the authors mentioned 37 genes with differential expression from adult males. Is it the total of 36 DEGs from this group?

Response: Thank you for pointing out this typo, it has been corrected.

2) What is the rationale for choosing a cafeteria diet over a readymade high-fat diet for rats?

Response: Our lab has used western-style 'cafeteria' diets to model diet-induced obesity in rats for many years. This model, which involves a rotating daily assortment of supermarket foods high in fat, sugar and salt, which incorporates palatability and variety leads to a more robust obesity phenotype than purified homogeneous high-fat diets (Buyukdere et al., 2019; Sampey et al., 2011; Leigh, Kendig & Morris, 2019). The cafeteria style and its variants more accurately model the human condition by encouraging a chronic positive energy balance - and subsequent obesity - through long term overconsumption of palatable, energy-dense foods. Thus we chose the cafeteria diet model for its ecological validity and the strong metabolic phenotype that it produces.

References

- Buyukdere, Y., Gulec, A., & Akyol, A. (2019). Cafeteria diet increased adiposity in comparison to high fat diet in young male rats. PeerJ, 7, e6656.
- Sampey, B. P., Vanhoose, A. M., Winfield, H. M., Freemerman, A. J., Muehlbauer, M. J., Fueger, P. T., ... & Makowski, L. (2011). Cafeteria diet is a robust model of human metabolic syndrome with liver and adipose inflammation: comparison to high-fat diet. Obesity, 19(6), 1109-1117.
- Leigh, S. J., Kendig, M. D., & Morris, M. J. (2019). Palatable western-style cafeteria diet as a reliable method for modeling diet-induced obesity in rodents. JoVE (Journal of Visualized Experiments), (153), e60262.

We have added the following to the Methods section lines 417-419:

“The ‘cafeteria’ style diet is high in fat and sugar (Leigh, Kendig & Morris, 2019) which leads to a more robust obesity phenotype than purified homogeneous high-fat diets (Buyukdere et al., 2019; Sampey et al., 2011).”

3) How is normalization performed for RNA-seq analysis?

© 2026. Published by The Company of Biologists under the terms of the Creative Commons Attribution License (https://creativecommons.org/licenses/by/4.0/).

Response: This is addressed in the response to the Major Concern 1: Normalisation was done with the Trimmed Mean of M-values (TMM) method.

Text added to lines 444-446 in the methods section: “Differential gene expression (DEG) analysis was performed using the Bioconductor packages, edgeR (v 3.38.4)(Robinson et al., 2010) and voom (limma)(Ritchie et al., 2015), using raw count data as input with normalisation performed using the Trimmed Mean of M-values (TMM) method.”

4) In the Figure 1 legends, authors should mention where the DEGs are coming from? Are these DEGs common in maternal obesity in both male and female weaner offspring?

Response: Thanks for raising this point - the legend has been amended and now reads:

“Heatmap showing the most significantly enriched KEGG pathways that were commonly up- or downregulated by maternal obesity in both male and female weaner offspring based on liver RNA-seq. Selected DEGs in each pathway are displayed.”

5) I request the authors to submit the RNA-seq and bisulphite-seq raw data or the sequence files in GEO database.

Response: This was a major undertaking and took 3 months to achieve the coordination with the NCBI, and transfer of 0.64Tb of data. 32 RRBS fastq files in 18 BioSamples, and 320 RNA-seq fastq files in 80 BioSamples are published under the NIH NLM BioProject PRJNA1475547.

Line 500 “All raw data files are available under the NIH NLM BioProject PRJNA1475547.”

***Reviewer 2**: Youngson et al. ask whether the male-specific postnatal liver demethylation program described in mice born to obese mothers is conserved in rats. The study combines RRBS and RNA-seq and reports a striking male-specific increase in DNA methylation at weaning in offspring of Caf-fed (obese) dams.*

The manuscript is interesting, but there are some points that need to be addressed.

1. There is an issue in how the findings are described. The abstract and framing refer to "increased genome-wide demethylation," whereas the reported data indicate that the vast majority of DMRs in males are hypermethylated. As written, this is logically inconsistent. It would be useful to clarify whether the authors intend to describe a reduction or failure of normal demethylation, rather than increased demethylation per se, and to ensure consistent terminology throughout the manuscript. Basically, the direction of methylation needs to be clearly stated.

Response: Thank you to the reviewer, for pointing out this poor phrasing, which was a mistake. Apologies for the confusion it caused. This has been made more clear in the abstract (line 45) and discussion lines 335-336 and 346-348.

Abstract: “Comparison of DNA methylation in 3-week-old weaner males versus female siblings from Chow-fed dams did not reveal the male-specific DNA demethylation that was previously reported in mouse. However, strong maternal diet effects in male weaner offspring methylation were observed. “

Discussion: “In male offspring almost all DMRs had higher methylation levels if they had a Caf-fed mothers than if they had a chow-fed mother. “

Discussion: “Instead our data suggest that in rat maternal Caf-diet, increases DNA methylation levels at thousands of regions throughout the genome.”

2. All methylation analyses are conducted at a single developmental stage (3 weeks). Given that the mouse demethylation process spans a broader postnatal window, the absence of a comparable pattern in the rat may reflect developmental timing rather than disruption. This possibility should be stated.

© 2026. Published by The Company of Biologists under the terms of the Creative Commons Attribution License (https://creativecommons.org/licenses/by/4.0/).

Response: We agree with this alternative explanation and have stated it in lines 342-345 "This may reflect a species-specificity of male postnatal DNA methylation in liver, or could suggest that it occurs at a different timepoint in rat. In mouse, the demethylation mainly occurs between 3 and 6 weeks after birth(Reizel et al., 2018), so may not have initiated in the 3-week old rats used in this study.".

3. The overlap between DMRs and DEGs is limited, particularly in males. While this is acknowledged, the manuscript still implies functional consequences that are not strongly supported by the data. Additionally, the first time DMR is mentioned, it should be defined.

Response: We agree that our main message is that the maternal-diet induced methylation differences in male offspring are not driving gene expression changes. This is strengthened further with the addition of **Supplementary Table 2** (see response to Reviewer 1's point 3) and **Supplementary Figure 4**.

We have strengthened the statement in the abstract lines 51-52 to state that "However, comparison with RNA-seq data revealed limited transcriptional changes at this developmental stage or in adult offspring."

DMR has been defined in the abstract and line 221.

4. KEGG pathway enrichment results are presented; however, none of the identified pathways remained significant after multiple testing correction. Despite this, these pathways are discussed in a biological context. These findings could be suggestive, as there is not robust support for mechanism.

Response: We apologise the the lack of clarity. It is only in the section '**Comparison of DMRs to regions undergoing post-natal demethylation in mouse liver**' that all the KEGG pathways did not pass multiple correction. Importantly, all KEGG pathways in Figure 1 remained significant even with both FDR and Benjamini multiple testing correction in both male and females.

<u>Second decision letter</u>

MS ID#: bio.062573R1

MS Title: Maternal obesity in rats results in male-specific increases in genome-wide DNA methylation in postnatal offspring liver

Authors: Neil A. Youngson; Susan M. Corley; Kyoko Hasebe; Aynaz Tajaddini; Michael D. Kendig; Margaret J. Morris

I am happy to tell you that your manuscript has been accepted for publication in Biology Open, pending our standard publication integrity checks. It was accepted on 23rd July 2026.

© 2026. Published by The Company of Biologists under the terms of the Creative Commons Attribution License (https://creativecommons.org/licenses/by/4.0/).
